# Evaluation of Selenium Nanoparticles in Inducing Disease Resistance against Spot Blotch Disease and Promoting Growth in Wheat under Biotic Stress

**DOI:** 10.3390/plants12040761

**Published:** 2023-02-08

**Authors:** Muhammad Shahbaz, Abida Akram, Asma Mehak, Ehsan ul Haq, Noor Fatima, Gull Wareen, Betty Natalie Fitriatin, R. Z. Sayyed, Noshin Ilyas, Mohd Khalizan Sabullah

**Affiliations:** 1 Department of Botany, PMAS-Arid Agriculture University Rawalpindi, Rawalpindi 46300, Pakistan; 2 Department of Agronomy, PMAS-Arid Agriculture University Rawalpindi, Rawalpindi 46300, Pakistan; 3 Department of Botany, Lahore College for Women University, Lahore 54000, Pakistan; 4 Department of Biology, Faculty of Sciences, PMAS-Arid Agriculture University Rawalpindi, Rawalpindi 46300, Pakistan; 5 Department of Soil Sciences and Land Resources Management, Agriculture Faculty, Universitas Padjadjaran, Jatinangor 45363, Indonesia; 6 Asian PGPR Society for Sustainable Agriculture, Auburn Ventures, Auburn, AL 36830, USA; 7 Faculty of Science and Natural Resources, University Malaysia Sabah, Kota Kinabalu 88400, Sabah, Malaysia

**Keywords:** *Bipolaris sarokiniana*, biocontrol, green synthesis, nanobiotechnology, spot blotch, SeNPs, wheat

## Abstract

In the present study, SeNPs were synthesized using *Melia azedarach* leaf extracts and investigated for growth promotion in wheat under the biotic stress of spot blotch disease. The phytosynthesized SeNPs were characterized using UV-visible spectroscopy, scanning electron microscopy (SEM), energy-dispersive X-ray (EDX), and Fourier-transformed infrared spectroscopy (FTIR). The in vitro efficacy of different concentrations of phytosynthesized SeNPs (i.e., 100 μg/mL, 150 μg/mL, 200 μg/mL, 250 μg/mL, and 300 μg/mL) was evaluated using the well diffusion method, which reported that 300 μg/mL showed maximum fungus growth inhibition. For in vivo study, different concentrations (10, 20, 30, and 40 mg/L) of SeNPs were applied exogenously to evaluate the morphological, physiological, and biochemical parameters under control conditions and determine when infection was induced. Among all treatments, 30 mg/L of SeNPs performed well and increased the plant height by 2.34% compared to the control and 30.7% more than fungus-inoculated wheat. Similarly, fresh plant weight and dry weight increased by 17.35% and 13.43% over the control and 20.34% and 52.48% over the fungus-treated wheat, respectively. In leaf surface area and root length, our findings were 50.11% and 10.37% higher than the control and 40% and 71% higher than diseased wheat, respectively. Plant physiological parameters i.e., chlorophyll a, chlorophyll b, and total chlorophyll content, were increased 14, 133, and 16.1 times over the control and 157, 253, and 42 times over the pathogen-inoculated wheat, respectively. Our findings regarding carotenoid content, relative water content, and the membrane stability index were 29-, 49-, and 81-fold higher than the control and 187-, 63-, and 48-fold higher than the negative control, respectively. In the case of plant biochemical parameters, proline, sugar, flavonoids, and phenolic contents were recorded at 6, 287, 11, and 34 times higher than the control and 32, 107, 33, and 4 times more than fungus-inoculated wheat, respectively. This study is considered the first biocompatible approach to evaluate the potential of green-synthesized SeNPs as growth-promoting substances in wheat under the spot blotch stress and effective management strategy to inhibit fungal growth.

## 1. Introduction

Emerging fungal and oomycete infections affect commercially significant commodity crops and staple calorie crops, posing a severe threat to global food security [1]. Wheat is among the top ten most popular and commonly cultivated crops worldwide [2]. Although wheat is Pakistan’s most important crop, contributing 10% of the country’s agricultural value and 2.2% of the GDP, the nation still struggles to meet the world average for wheat output [3]. Different regions of the world experience losses in wheat output due to various diseases. Spot blotch disease is caused by *Bipolaris sarokiniana*, which affects wheat varieties in numerous regions of the world and causes significant yield losses [4]. In Pakistan, spot blotch disease has a crucial effect on wheat production among all major diseases of wheat, including rusts, smut, and powdery mildew [5]. Almost all wheat-growing regions are affected by this disease [6]. Devi et al. reported that in warmer areas of wheat cultivation, this pathogen becomes resistant, and 16%–43% of wheat loss occurs in warmer regions due to *B. sarokiniana* [7]. Utilizing fungicides is one of the most well-known ways to treat fungus problems but is damaging to human health and causes collateral damage [8].

Biological control is an alternative way of reducing the use of chemicals in agriculture. Using plant growth-promoting rhizobacteria (PGPR) as plant growth and health-stimulating agents is a biological method [9]. PGPR are soil-borne bacteria that colonize the roots of plants and play a significant role in the protection and growth of plants in several ways. They promote plant growth either directly through the production of hormones or indirectly through the production of antimicrobial compounds which act against pathogens, viz., induced systemic resistance in the host, antibiotic production, growth promotion, and competition of nutrients [10]. The application of PGPR as a biocontrol agent against fungal pathogens showed promising results in greenhouse systems. However, the environmental conditions in the greenhouse are consistent throughout the season of crop plants. Achieving such constant environmental conditions in the field is not possible, where the variability of abiotic and biotic factors is higher, and competition with indigenous organisms is more stressful [11]. Some modern technology can be used in agriculture to develop innovative techniques that reduce the usage of harmful agrochemicals and help increase productivity. Nanotechnology is the advanced science of the 21st century; creating materials in nanoform (1–100 nm) has many applications in plant protection and disease management [12]. For the control of plant diseases and improvement of growth-related parameters, nanoparticles have been used in place of bactericides/fungicides and nano-fertilizers [13].

Selenium is an essential element as well as a micronutrient that has been frequently used in the treatment of diseases [14]. Selenium in nano form has safer and more cost-effective antibacterial and antioxidant effects than in its other forms [15]. Using different SeNPs concentrations (100, 200, and 250 µg/mL), the antimicrobial activity of SeNPs against various phytopathogenic bacteria and fungi was determined. However, 100 µg/mL showed the best results in controlling 99 percent of different bacteria, including *Pseudomonas aeruginosa*, *Staphylococcus aureus*, and *Escherichia coli.* Utilizing selenium nanoparticles (SeNPs), which are less toxic and have higher bioavailability and biological activity, is a novel method of fertilizing plants [16]. SeNPs enhanced the phenolic compounds, total chlorophyll contents, antioxidant defense systems, and morphological and genetical attributes of the *Vicia faba* plant under biotic stress of *Rhizoctonia solani*. [17]. The antifungal potential of plant-based SeNPs at 100 ppm is the most effective to inhibit the growth of *Alternaria alternata*, which causes leaf blight in tomatoes [18].

SeNPs have been synthesized using a variety of techniques. However, reducing chemicals such as hydrazine and sodium ascorbate were employed chemically to synthesize SeNPs to create their multi-functional potential. The chemical processes are expensive, demand specialized equipment, and are damaging to the environment. Synthesis of selenium nanoparticles is an eco-friendly, non-toxic, biocompatible, and cheap means of synthesizing NPs because plant extracts act as reducing and stabilizing agents [19]. The utilization of green nanotechnology is sustainable and practical and replaces the usage of hazardous chemicals. Plants absorb green SeNPs 20 times more quickly than bulk selenium. SeNPs are produced by various plants, notably those that do not utilize selenium. *Aloe vera*, *Withania somnifera*, *Diospyros Montana*, and *Trigonella foenum-graecum* are a few examples [20]. The main objective of the current study is to develop a novel protocol of plant-based SeNPs that would promote the growth, physiological, and biochemical parameters of wheat under biotic stress of spot blotch disease under in vivo conditions and develop antifungal potential against *B. sarokiniana* under in vitro study.

## 2. Materials and Methods

### 2.1. Preparation of Plant Extract

The plant extract was prepared using the methodology of Fardsadegh-Jafarizadeh-Malmiri [21]. Fresh and green leaves of *M. azedarach* were obtained from PMAS-Arid Agriculture University, Rawalpindi, Pakistan. Leaves were washed to remove impurities such as dust particles and dried for a week at room temperature. After drying, the leaves were ground with a grinder to obtain fine powder; 4.69 g of this powder was put into 100 mL of distilled water and allowed to boil for 5 min to obtain the leaf extract. After this, the extract was filtered through Whatman no.1 filter paper three times to obtain the pure extract. Sterile conditions were maintained in each step of the experiment to obtain contaminate-free and accurate results.

### 2.2. Synthesis of Selenium Nanoparticles

SeNPs were synthesized by mixing the plant extracts with a salt stock solution. The stock solutions of sodium selenite (10 mM) were prepared by adding 1.25 g of sodium selenite salt to 500 mL of distilled water and then allowed to heat at 80 °C along with magnetic stirring on a hot plate (Sr. No G150) for 30 min. Then, 150 mL of plant extract was added dropwise into the stock solution until its color changed from green to brick-red after 2 h of continuous heating and magnetic stirring. When the brick-red color formed, it was then allowed to cool. Centrifugation was performed using a centrifuge machine (Z 326 CA, Hermle, Franklin, WI, USA) at 1000 rpm for 15 min at 25 °C. The supernatant was discarded, and the pellet was collected by adding methanol. After it was collected, the pellet was centrifuged thrice to remove plant extracts and salt [22]. The resulting SeNPs were subjected to characterization and then were used for in vitro and in vivo purposes.

### 2.3. Characterization of Selenium Nanoparticles

Green synthesized SeNPs were characterized using different characterization techniques, i.e., UV-visible spectroscopy, SEM, EDX, XRD, and FTIR. Formation of SeNPs from sodium selenite was seen using UV-visible spectroscopy. The generated SeNPs were mixed with sterile water and ultra-sonicated for 5–10 min. The spectrum was recorded from 200 to 800 nm for UV-visible spectroscopy analysis [23]. SEM was used to evaluate the structural analysis of the produced SeNPs with the help of the SIGMA model operated at 5 KV, enlargement 10 K, from the Institute of Space and Technology (IST), Islamabad. After that, a film of the sample was formed on a carbon-coated copper grid by simply reducing the SeNPs into a water suspension on the grid; excess solution was removed using the sample film that formed on a carbon solution with blotting paper and allowing the film on the SEM grid to dry for five minutes under a mercury lamp. Surface photos of the samples were captured at various magnifications. An elemental analysis of green-produced SeNPs was performed using an energy-dispersive X-ray (EDX) detector after the NPs were dropped on the film of carbon. The crystalline nature of the SeNPs was investigated using an XRD spectroscopy technique. Powdered samples were utilized for analysis of the diffraction pattern on a Shimadzu model XRD 6000 JP US in the 5°–50° in 2*θ* angle range. The Debye–Scherrer equation was used to compute the average size of the synthesized SeNPs,

(1) $$D=\frac{k\lambda }{\beta D}$$

where *k* describes the shape factor, *λ* relates to the X-ray wavelength, *β* refers to the full width in radians at the highest point, and *θ* is the Bragg’s angle.

FT-IR was used to investigate the functional groupings (Perkin Elmer Spectrum 100 FT-IR Spectrometer WLM USA). The IR (infrared) spectra were recorded at a resolution of 4.0 cm^−1^ in the middle wavelength range of 4000−400 cm^−1^.

### 2.4. In Vitro Antifungal Assay

#### 2.4.1. Sample Collection and Isolation and Identification of Fungus

Leaves infected with spot blotch disease were collected from the village of Ghari Kandi in the district of Bahawalpur, Punjab, Pakistan (longitude: 71.286928, latitude: 29.293757). The leaves were surface-sterilized with 2% sodium hypochlorite and 70% ethanol three times, and leaves were cut into small pieces and placed in a Petri plate containing PDA media, which was prepared by adding 39 g of potato dextrose agar (Neogen) to 100 mL of distilled water, autoclaved for 15 min at 121 °C. The antibacterial drug clindamycin (0.2 mL/100 mL) was added into the PDA media. The Petri plates were covered with parafilm tape and then placed in an incubator at 28 °C for five days until the fungus started to grow. A similar methodology was followed to obtain a pure culture of fungus by placing fungus spore cultures using forceps into Petri plates containing PDA media. A pure culture was obtained after 4 days of incubation at 28 °C in an incubator [24].

With the help of an inoculating needle and a stereoscope, one *Bipolaris sarokiniana* spore was placed in a potato dextrose agar medium to purify the fungus [25]. Using a light microscope, slides were made and examined at 40x and 100x magnifications. The spore’s size and shape were measured according to Raza et al. [26]. When it was grown on PDA, several traits were noticed. Color, growth, and other characteristics of fungi were observed (Supplementary Figure S1).

#### 2.4.2. Evaluation of Antifungal Activity of SeNP-Well Diffusion Assay

The potato dextrose agar was prepared by dissolving 39 g of prepared PDA media (Neogen), autoclaved at 121 °C, and pouring it into Petri plates. After solidification, 3 wells were constructed using a metallic borer, each of 5 mm diameter; then the fungus spores’ suspension (1.5 × 10^8^ CFU/mL, calculated via hemocytometer) was spread on the whole plate using a glass rod. Antifungal Barresten tablets (500 mg) were purchased from a local pharmacy (Double Road, Rawalpindi), and 200 µg/mL of their concentration was placed into the wells for positive control. The wells were tagged with antifungal drugs with a concentration of 200 µg/mL, and different concentrations of SeNPs were used ranging from 100 µg/mL, 150 µg/mL, 200 µg/mL, 250 µg/mL, and 300 µg/mL respectively, using the methodology of Abdel-Moneim et al. [27] with little modification. The solution, 100 µL, was put into each well. The Petri plates were incubated for 1 day at 37 °C and for 5 days at 28 °C. The inhibition zone diameter was noted using the ordinary scale described by Menon et al. [28].

### 2.5. In Vivo Activity

#### 2.5.1. Greenhouse Experiment

A greenhouse experiment was conducted to determine the antifungal potential against *B. sarokiniana* (the causing agent of spot blotch disease in wheat) through the foliar spray of plant-based SeNPs. Earthen pots having a soil capacity of 10 kg were filled with sterilized soil. For the greenhouse experiment, a sandy loam soil was preferred. The composition of the soil was such that it contained clay (40%), silt (20%), and sand (40%). The wheat variety Galaxy- 2013 (disease-susceptible) was taken from the National Agriculture Research Center, Islamabad (NARC). The methodology of Abdel-Moneim et al. [27] was followed for surface sterilization of seeds using 0.1% of mercuric chloride. In the beginning, a completely randomized design (CRD) was set in triplicate form. First of all, low concentrations of SeNPs were used in a foliar application, then were gradually increased to evaluate the impact of plant-based SeNPs against *B. sarokiniana* (Table 1).

#### 2.5.2. Fungal Inoculum’s Preparation and Application

The fungal pathogen *B. sarokiniana* was isolated from infected wheat plants and then grown using the potato dextrose agar (PDA) media. The maximum fungal growth was achieved by placing inoculated agar plates into an incubator at 23 °C for 10 days. By scrubbing 12 day-old cultures and mixing them with autoclaved distilled water, fungal inoculums were prepared and placed in an orbital shaker at room temperature for pure conidial cultures. The hemocytometer was used to adjust to 8000 conidia per ml. Tween 20 was used to disperse the conidia, as reported by Satti et al. [29]. At the booting stage of wheat, the fungal inoculum was applied directly onto the leaves, and each plant received 50 mL of suspension as a fine mist using a pressurized atomizer. Spores were inoculated into the greenhouse using a mister to keep the temperature at 18 °C, and an 80 to 90% moisture level was adjusted. For successful pathogen propagation, the plants were covered with clear polythene bags and repeatedly sprayed with autoclaved water after being sprayed with the spore solution. The temperature was set at 16–18 °C. The first data were recorded on the 5th day after inoculation and then after every 10th day (day 15, day 20, day 25, and day 30).

#### 2.5.3. Evaluation of the Disease Incidence

The leaves were taken randomly from different pots in triplicate form. The symptoms revealed the severity of spot blotch disease caused by *B. sarokiniana* and were observed on a visual basis (Table 2).

The disease severity was analyzed on a standard scale of 0–5 for disease as described by Iftikhar et al. [27]. By following the equation of Iftikhar et al. [30], disease incidence was determined.

(2) $$\mathrm{Disease}\;\mathrm{incidence}\;\left(\%\right)=\frac{\mathrm{Number}\;\mathrm{of}\;\mathrm{infected}\;\mathrm{plants}}{\mathrm{Total}\;\mathrm{number}\;\mathrm{of}\;\mathrm{Plants}}\times 100$$

The disease severity was also measured by following the formula given by Iftikhar et al. [30].

(3) $$\mathrm{Percent}\;\mathrm{Disease}\;\mathrm{Index}\;\left(\mathrm{PDI}\right)=\frac{\mathrm{Disease}\;\mathrm{index}}{\mathrm{Total}\;\mathrm{Infected}\;\mathrm{Plants}}\times 100$$

Disease index = (Spot in scale 1) + (Spot in scale 2) + …… (Spot in scale 5)(4)

#### 2.5.4. Collection of Samples

By using a random sampling technique from three pots of each treatment, samples were taken to evaluate the plant growth parameters (i.e., plant height, fresh plant weight, dry plant weight, and root length), physiological parameters (i.e., chlorophyll contents, carotenoid contents, relative water contents, and membrane stability index), biochemical parameters (i.e., Proline content, sugar content, total phenolic and flavonoid content under biotic stress), and the control condition.

### 2.6. Plant-Growth Promotion Study

Plant height was measured using an ordinary scale. The leaf surface area was determined using a leaf area meter (CID, Bioscience, Inc.US) when the leaves were at the flag-leaf stage. The fresh weight of shoots and roots was analyzed for each treatment, and the same plants were dried in a hot-air oven for one week at 65 °C to record the dry weight, according to Iqbal et al. [31].

### 2.7. Analysis of Plant Physiological Parameters

#### 2.7.1. Chlorophyll and Carotenoid Content

The chlorophyll content of the leaf was measured using a spectrophotometer (Model U-2900 Sr. No 26E82-018 Hitachi High-Tech Global JP). The plant leaf (2 g) was ground with 10 mL of acetone and was filtered thrice in a test tube, and absorbance was observed at 645 nm, 652 nm, and 663 nm wavelengths. For carotenoid content, a similar methodology was performed, but absorbance was taken at 480 nm as per Bruinsma et al. [32]. The following equation was used to calculate the chlorophyll content of the leaf:

Ch a = 12.7(A_663_) − 2.7(A_645_) (5)

Ch b = 22.9(A_645_) − 4.7(A _663_) (6)

Ch total = (A_652_ ×1000/34.5) (7)

Mullan and Pietragalla [33] described the methodology to determine the relative water content of the leaves. For the fresh-weight leaf measurement, leaves were put into Petri plates containing water. After 24 h, again, the leaf weight estimated is the turgid weight (TW). After measuring turgid weight, the leaves were packed into small packets and put into the oven at 70 °C for one week. After seven days, the weight of the leaves was measured, which was the dry weight (DW). Using the following formula, the relative water content (RWC) of the leaves was measured:

(8) $$\left(\%\right)\;\mathrm{Relative}\;\mathrm{water}\;\mathrm{content}\;\left(\mathrm{RWC}\right)=\frac{\mathrm{Fresh}\;\mathrm{weight}-\;\mathrm{Dry}\;\mathrm{weight}}{\mathrm{TW}\;–\mathrm{Dry}\;\mathrm{weight}}\times 100$$

#### 2.7.2. Membrane Stability Index (%)

A leaf from each sample was taken and cut into small discs (100 mg) and washed with distilled water; then, discs were put into test tubes, and the test tubes were placed into a water bath for 30 min at 40 °C. The (C1) electrical conductivity was then measured using an EC meter. The electrical conductivity (C2) of the test tubes was then measured after ten minutes in the water bath at 100 °C using the methodology of Sairam et al. [34].

A formula is used to calculate the MSI%:

(9) $$\%\;\mathrm{MSI}\;=1-\frac{\left(C1\right)}{\;\left(C2\right)}$$

### 2.8. Assessment of Plant Biochemical Parameters

#### 2.8.1. Proline Content

Sulfosalicylic acid (4 mL) (3%) was used for crushing fresh leaves (0.2 g), and 2 mL was placed in separate test tubes and allowed to react with the ninhydrin component and 2 mL of frozen acetic acid. It was boiled in water and, after the formation of a color response, clogged by putting the test in a freezer. After that, it was added to 4 mL of toluene and mixed thoroughly until the higher colorful layer appeared. This layer separated from the rest of the liquid in another set of test tubes, and absorbance was measured at 520 nm according to the methodology of Bates et al. [35]. Proline content was calculated using the formula below.

(10) $$\mathrm{TPC}\;\left(\text{µ}\frac{g}{\mathrm{mL}}\right)=\frac{\mathrm{Sample}\;\mathrm{absorbance}\;\times \;\mathrm{Dilution}\;\mathrm{factor}\times \;K\;\mathrm{value}}{\mathrm{Fresh}\;\mathrm{weight}\;\mathrm{of}\;\mathrm{plant}\;\mathrm{tissue}}$$

#### 2.8.2. Soluble Sugar

According to Qayyum et al. [36] from fresh leaves, 0.5 g was taken and mixed in 10 mL of 80% ethanol; after that, it was boiled for one hour at 80 °C in a water bath. One ml of phenol (18%) and 0.5 mL of extract were combined in test tubes and left to incubate at room temperature after mixing and shacking one 2.5 mL of sulfuric acid correctly., Using a spectrophotometer (Model U-2900 Sr. No 26E82-018 JP), the absorbance of each duplicate was checked at a 490 nm wavelength.

(11) $$\mathrm{Soluble}\;\mathrm{Sugar}\;=\frac{\mathrm{Sample}\;\mathrm{absorbance}\;\times \;\mathrm{Dilution}\;\mathrm{factor}\;\times \;K\;\mathrm{value}}{\mathrm{Weight}\;\mathrm{of}\;\mathrm{Fresh}\;\mathrm{Plant}\;\mathrm{Tissue}}$$

#### 2.8.3. Total Phenolic Content

By following the methodology of Hussein et al. [37], total phenolic content was determined. A total of 0.75 mL of the Folin–Ciocalteu reagent was combined with 100 mL of plant extract and incubated for 5 min at 22 °C. The mixture was then given 0.75 mL of Na_2_CO_3_ solution and kept at 22 °C for 90 min. Finally, the sample’s absorbance at 725 nm was measured using a UV–visible spectrophotometer (Model U-2900 Sr. No 26E82-018 JP).

#### 2.8.4. Total Flavonoid Content

By using the protocol of Hussein et al. [37], the total flavonoid content was analyzed. Quercetin (10 mg) was dissolved in 80% ethanol and diluted several times. The resultant standard solution was combined with 0.1 mL of 1 M potassium acetate, 0.1 mL of 10% aluminum chloride, 1.5 mL of 95% ethanol, and 2.8 mL of distilled water, and then incubated at room temperature for 30 min. Then, using a UV-visible spectrophotometer (Model U-2900 Sr. No 26E82-018 JP), the absorbance of the combination was measured at 415 nm.

## 3. Results

### 3.1. Green Synthesis and Characterization of Selenium Nanoparticles

The physical change in solution color from light green to brick red confirmed the formation of SeNPs (Supplementary Figure S2).

**Figure 1:**
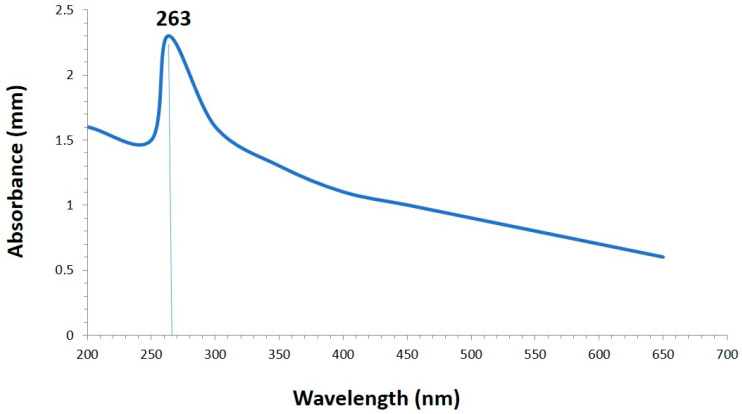
UV-visible spectroscopy of plant-based selenium nanoparticles.

**Figure 2:**
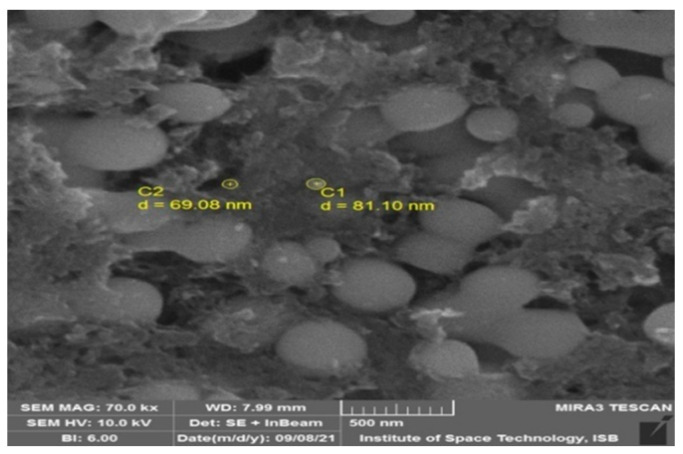
Scanning electron microscopy of SeNPs.

**Figure 3:**
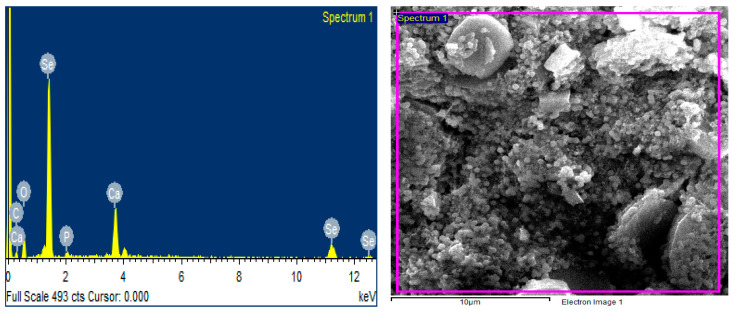
EDX Analysis of Plant based selenium nanoparticles.

**Figure 4:**
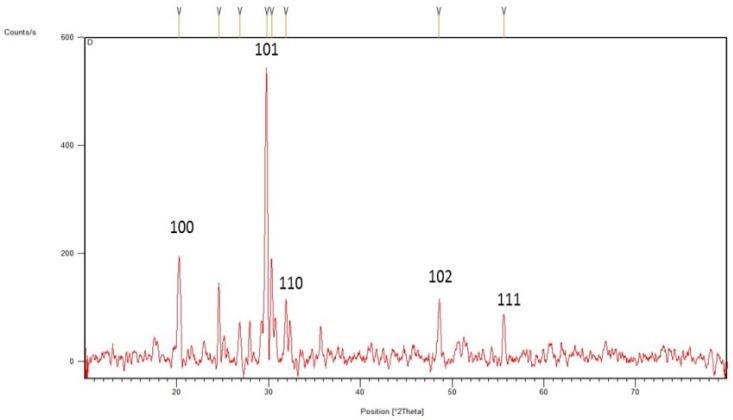
XRD analysis of selenium nanoparticles.

**Figure 5:**
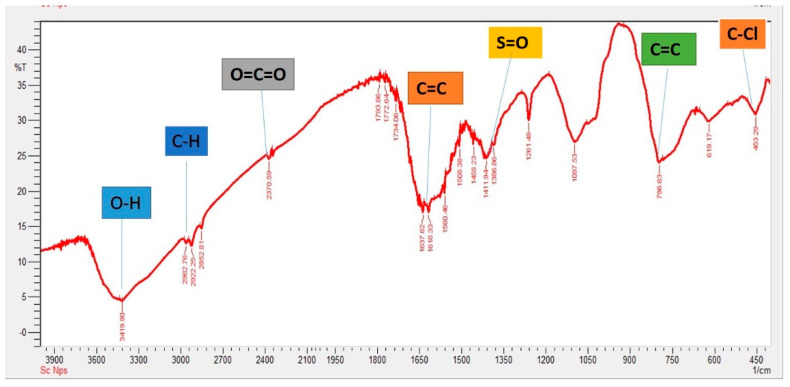
FTIR analysis of SeNPs.

The synthesis of SeNPs was confirmed using different characterization techniques, and visible spectroscopy revealed that a clear peak was formed at 263 nm, which confirmed the SeNPs as shown in Figure 1. The morphology of green-synthesized SeNPs was confirmed using SEM, which revealed the spherical shape with an average size of approximately 74.43 nm (Figure 2). According to the SEM image, some SeNPs were spherical in shape while a few have an irregular shape. An EDX detector was used to confirm the presence of metallic selenium ions. The EDX spectrum elucidated strong absorption peaks of metallic selenium ions at 1.35 KeV, 11.20 KeV, and 12.40 KeV (Figure 3). The EDX analysis revealed that selenium also exists in elemental form along with other elements in the form of peaks. The crystalline nature of SeNPs was confirmed using XRD. The SeNPs X-ray plans (100), (110), (101), (111), (102) were matched at diffraction peaks at 2Ɵ of 20.25°, 24.593°, 26.862°, 29.809, and 30.327° (Figure 4). By analyzing the chemical bond vibration rates, the Fourier transform infrared spectrometer (FTIR) reveals the functional groups present on the surface of the NPs. The complete spectra of *M. azedarach* leaf extract and biosynthesized SeNPs are shown in Figure 5. The absorption peak of 3419.90 cm^−1^ corresponds to the hydroxyl group (O–H). The absorption peaks of 2962.76 cm^−1^, 2922.25 cm^−1^, and 2852.81 cm^−1^ represent the occurrence of carbon–hydrogen stretching (C–H). Similarly, the peak at 2370.58 cm^−1^ confirmed the presence of carbon dioxide (0=C=0). The absorption peaks in 1793.86 cm^−1^, 1772.64 cm^−1^, and 1734.06 cm^−1^ represent the carboxyl groups. The absorption peaks at 1637.62 cm^−1^ and 1618.33 cm^−1^ relate to C=C. The absorption peaks at 1560.46 cm^−1^ and 1508.38 cm^−1^ relate to N–O, and at 1458.23 cm^−1^ relates to C–H. The absorption peaks at 1411.94 cm^−1^ and 1386.86 cm^−1^ relate to S=O. The absorption peaks at 1261.49 cm^−1^ and 1097.53 cm^−1^ correspond to C–O. The absorption peaks at 796.63 cm^−1^, 619.17 cm^−1^, and 453.29 cm^−1^ confirmed the presence of C=C, C-Br, and C-Cl, respectively.

### 3.2. Morphological and Microscopic Identifications

After isolation of the fungus from the infected wheat variety, the pathogen was physically observed due to its colony growth and color. The grayish-black colony was observed and matured after 4–6 days; a suppressed type of growth was observed.

#### 3.2.1. Antifungal Assay (Well Diffusion Method)

**Figure 6:**
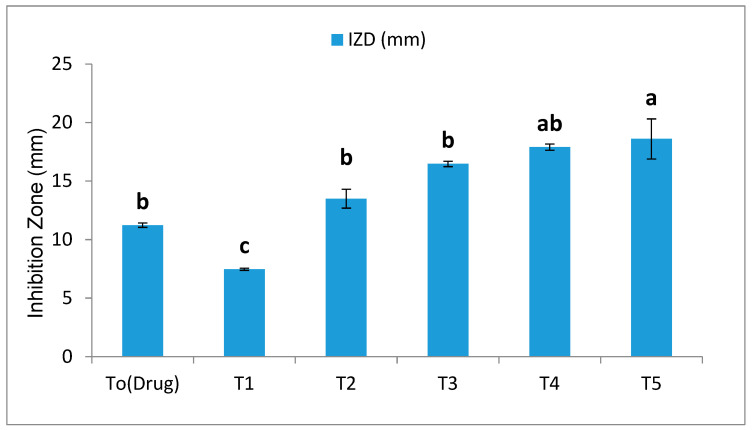
Application of different concentrations of biogenic selenium nanoparticles against *B. sarokiniana* causing agent of spot blotch disease in wheat using well diffusion method. Mean ± SE; n = 3. Different alphabetical letters on bars statistically significant variation at *p* < 0.05 as per DMRT.

The antifungal activity of SeNPs was evaluated against *B. sarokiniana* using the agar well diffusion method at different concentrations, i.e., 100, 150, 200, 250, and 300 μg/mL, and the results were compared with the antifungal drug Barresten (standard). The results presented in Figure 6 reveal that by increasing the concentrations of SeNPs, the inhibition zone diameter increased. The antifungal drug (Barresten) produced a diameter of 11.233 mm. Similarly, the lowest and the highest inhibition zone diameters were produced by T1 and T5, i.e., 7.467 mm and 18.60 mm, respectively (Supplementary Figure S3). Among all treatments, T5 = 300 µg/mL is the most appropriate concentration of SeNPs that exhibited a maximum inhibition zone diameter compared to the recommended antifungal drug (Barresten). Therefore, it was proved that biofabricated SeNPs with a concentration of 300 µg/mL possess good antifungal activity when compared to antifungal drugs.

#### 3.2.2. Assessment of Disease Incidence (%)

**Figure 7:**
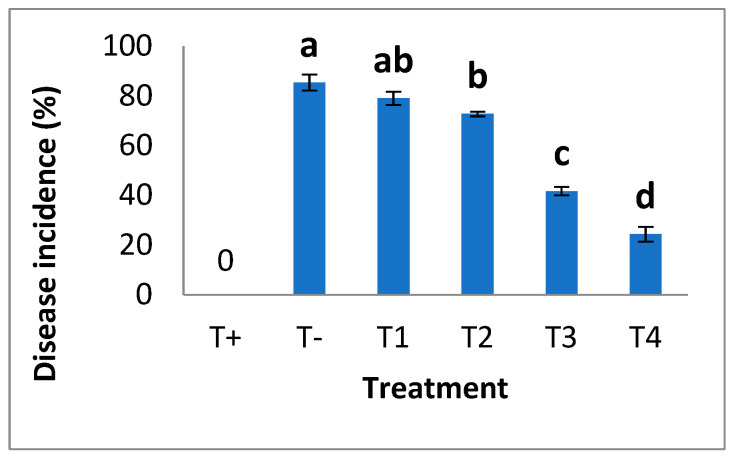
Disease incidence (%) in response to applications of different concentrations of SeNPs. Mean ± SE; n = 3. Different alphabetical letters on bars statistically significant variation at *p* < 0.05 as per DMRT.

Visual symptoms of disease measured disease incidence. It was observed that the disease index was maximized in plants with no NPs; however, the severity of disease symptoms decreased by the foliar application of different concentrations of bio fabricated SeNPs. Minimum disease symptoms were recorded in plants treated with 30 mg/L and 40 mg/L of SeNPs (Figure 7).

#### 3.2.3. Effect of Biosynthesized Selenium Nanoparticles on Plant Morphological Aspects

**Figure 8:**
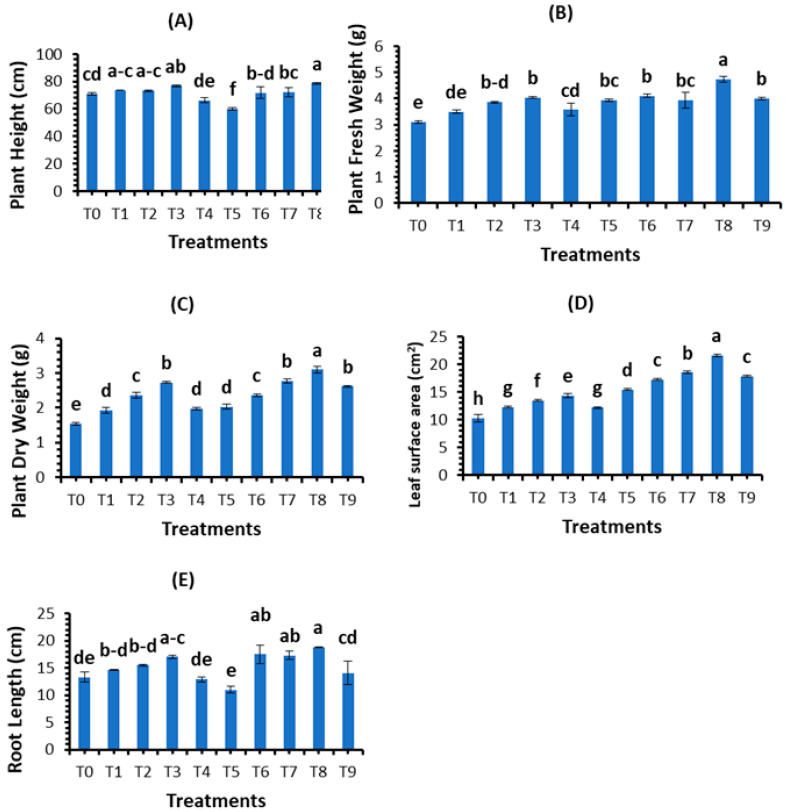
Effect of bio fabricated selenium nanoparticles on the morphological attributes: (**A**) plant height; (**B**) plant fresh weight; (**C**) plant dry weight; (**D**) leaf area; and (**E**) root length of wheat under biotic stress. Mean ± SE; n = 3. Different alphabetical letters on bars statistically significant variation at *p* < 0.05 as per DMRT.

The morphological parameters of wheat were investigated to analyze the potential of green-synthesized SeNPs against *B. sarokiniana* causing spot blotch disease. Different conc. (10, 20, 30, and 40 mg/L) of SeNPs were applied on control plants as well as on fungal-inoculated wheat plants as a foliar spray, and morphological parameters were recorded in terms of plant height, plant fresh weight, plant dry weight, leaf area, and root length. The findings revealed that all concentrations of SeNPs increased the growth parameters, i.e., plant height, plant fresh weight, plant dry weight, leaf area, and root length. At the concentration of 30 mg/L, plant height (76.9 cm), fresh plant weight (4.03 g), plant dry weight (2.73 g), leaf area (14.37 cm^2^), and root length (17.03 cm) increased significantly in control plants. The growth parameters were observed to decrease in fungal-inoculated plants. Under biotic stress, SeNPs also promoted the growth parameters. Among all treatments, 30 mg/L of SeNPs was the most appropriate concentration that boosted the plant height (78.63 cm), plant fresh weight (4.73 g), plant dry weight (3.1 g), leaf area (21.57 cm^2^), and root length (18.8 cm) (Figure 8).

### 3.3. Evaluation of Plant Physiological Parameters

**Figure 9:**
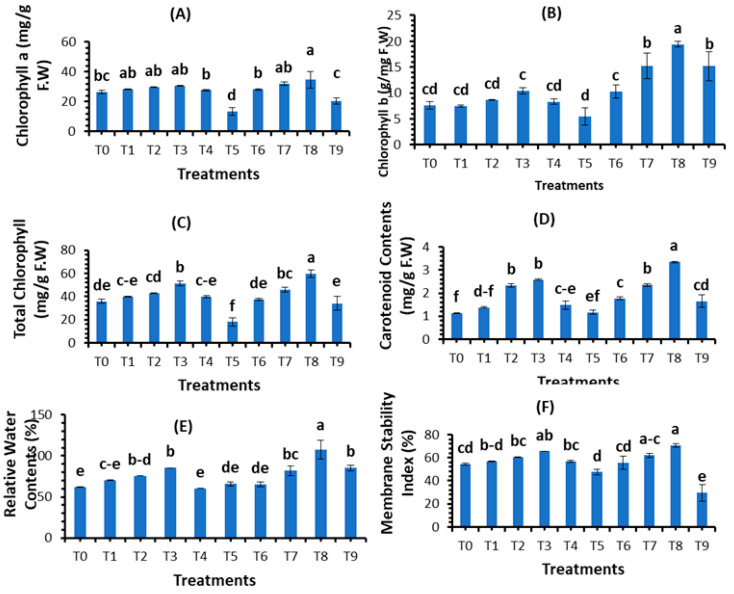
Application of different concentrations of SeNPs: (**A**) chlorophyll a; (**B**) chlorophyll b; (**C**) total chlorophyll; (**D**) carotenoid content; (**E**) relative water content; and (**F**) membrane stability index (%). Mean ± SE; n = 3. Different alphabetical letters on bars statistically significant variation at *p* < 0.05 as per DMRT.

The results presented in Figure 9 show the impact of phytosynthesized SeNPs on physiological attributes, i.e., chlorophyll a, chlorophyll b, total chlorophyll, carotenoid contents, relative water contents, and membrane stability index (%), using different concentrations of bio fabricated SeNPs against spot blotch disease of wheat. The results reveal that all physiological parameters were significantly promoted using a foliar application of SeNPs compared with control treatments and infected fungal treatments. According to our findings, 30 mg/L of SeNPs concentration significantly increased chlorophyll a (30.53 mg/g F.W), chlorophyll b (10.46 mg/g F.W), total chlorophyll (51.45 mg/g F.W), carotenoid content (2.50 mg/g F.W), relative water content (85.60%), and the membrane stability index (65.55%) in healthy plants, while in fungal inoculated wheat, the morphological parameters chlorophyll a (13.44 mg/g F.W), chlorophyll b (5.50 mg/g F.W), total chlorophyll (18.06 mg/g F.W), carotenoid content (1.17 mg/g F.W), relative water content (65.83%), and the membrane stability index (47.52%) decreased. The foliar application of SeNPs on fungal-inoculated wheat boosted the physiological parameters. Among all applied SeNPs treatments, T_8_ = 30 mg/L significantly promoted chlorophyll a (34.67 mg/g F.W), chlorophyll b (19.43 mg/g F.W), total chlorophyll (59.77 mg/g F.W), carotenoid content (3.35 mg/g F.W), relative water content (107.35%), and the membrane stability index (70.59%). Therefore, T_8_ = 30 mg/L is a suitable concentration that boosted the physiological parameters of the plant in both control and stress conditions.

### 3.4. Assessment of Plant Biochemical Parameters

**Figure 10:**
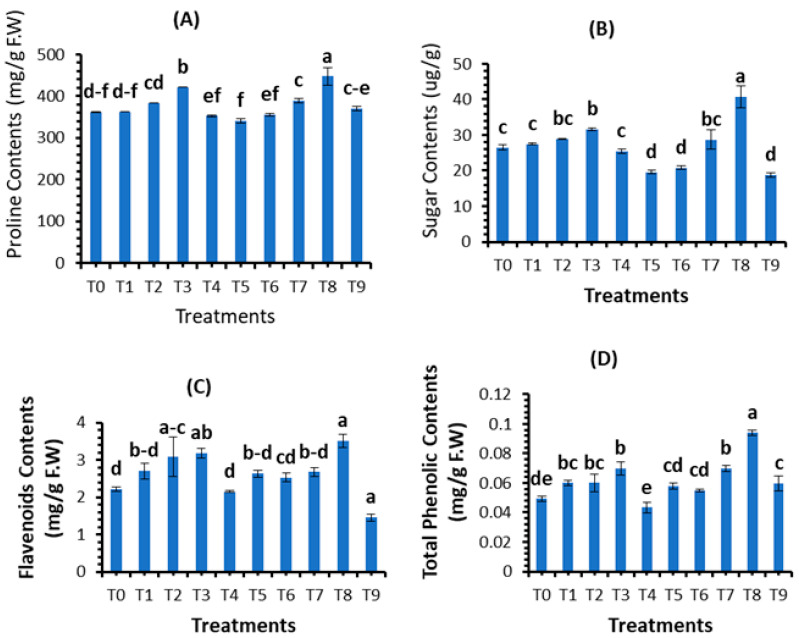
Effect of bio fabricated selenium nanoparticles on biochemical parameters: (**A**) proline content; (**B**) soluble sugar content; (**C**) flavonoid content; and (**D**) total phenolic content. Mean ± SE; n = 3. Different alphabetical letters on bars statistically significant variation at *p <* 0.05 as per DMRT.

The different concentrations (10, 20, 30, and 40 mg/L) of plant-based SeNPs were applied on healthy plants and on plants infected with *B. sarokiniana* and compared with positive and negative controls. The results showed that 30 mg/L of SeNPs concentration promoted the proline content (421.1 mg/g) and soluble sugar content (31.64) in control plants treated with SeNPs, which were higher when compared with the positive control. Similarly, the proline content (447.16 mg/g) and soluble sugar content (40.74 ug/g) recorded using 30 mg/L SeNPs were higher than the negative control (Figure 10A,B).

The data presented in Figure 10C,D show that total phenolic content (0.07 mg/g F.W) and total flavonoid content (3.177 mg/g F.W) were recorded higher using the foliar application of 30 mg/L of SeNPs when compared with the positive control, and 30 mg/L of SeNPs increased the total phenolic content (0.094 mg/g F.W) and total flavonoid content (3.51 mg/g F.W) when compared with negative control. Therefore, 30 mg/L of SeNPs is the most appropriate concentration that boosted the total phenolic and flavonoid contents both in normal and stress conditions.

## 4. Discussion

### 4.1. Synthesis and Characterization of SeNPs

The present work was performed both in vitro and in vivo to check the effect of phytosynthesized SeNPs on wheat under control conditions as well as under the biotic stress of *B. sarokiniana* (the causing agent of spot blotch disease in wheat) to evaluate the growth, physiological, and biochemical attributes of wheat.

The use of a biological mass such as plant extracts or plant biomass could be an alternative to physical and chemical methods for the synthesis of NPs in an eco-friendly manner that is safe, less time consuming, and low-cost [38]. In the present study, the phytosynthesis of SeNPs was carried out using *Melia azedarach* leaves as the main reducing and stabilizing agent. During the reduction reaction, the color of Na_2_SeO_3_ changed from colorless to brick-red, indicative of NP production [39]. The formation of the brick-red color was due to the fact that ingredients present in plant extracts could intermingle with selenite ions and reduce these ions into SeNPs [40]. Our observations were similar to those of Alsaggaf et al. [41], who synthesized the SeNPs using the leaf extract of *Ginkgo biloba*. Similarly, Satgurunathan et al. [22] used Na_2_SeO_3_ and *Allium sativum* clove extract for the formation of SeNPs. A UV-visible spectrophotometer reported the absorption peak at 263 nm. Surface plasmon resonance (SPR) is involved in the formation of absorbance peaks and confirms the formation of SeNPs. SPR is a resonance phenomenon induced by the interaction of conduction electrons of metal NPs with incoming photons [42]. Previous studies reported that phytosynthesized SeNPs have UV-visible maximum absorption in various ranges. Alagesan and Venugopal [43] and Anu et al. [44] produced SeNPs using garlic cloves and reported an absorption peak at 260 nm. Similarly, a few researchers have described the synthesis of SeNPs using various reductant agents [45,46,47].

The SeNPs were subjected to SEM to evaluate the size of the NPs. The resulting NPs possessed spherical shapes, and some were anisotropic. Our study agreed with Verma and Maheshwari [48], who reported a 74.25 nm average size of SeNPs. The findings were in line with Ikram et al. and Shahbaz et al. [49,50], who reported spherical SeNP production from plant extracts. The previous findings of Ezhuthupurakkal et al. and Fritea et al. [51,52], with the reported sizes of 205 nm and 50–100 nm, respectively, support our reported size. Researchers prepared SeNPs from leaf extracts of *Diospyros montana, Capsicum annuum*, and *Allium sativum*, and calculated SeNPs sizes of 80, 50–150, and 40–100 nm, respectively [53,54,55], which support the current findings.

SeNPs also contained reduced levels of O and C, which could be attributed to the flavonoids and phenolic content found in the *M. azederech* leaf extract. Gunti et al. [56] used *E. officinalis* fruit extract for SeNPs synthesis and described that SeNPs are chemically made of Se, O, and C, with Se shown at the highest peak. Matai et al. [57] used *Phyllanthus emblica* for SeNPs production, and showed that SeNPs are mostly composed of Se. Our findings also agreed with Menon et al. [58].

The reflection plans (100), (110), (101), (111), and (102) during XRD analysis showed that the diffraction peaks at 2 theta 20.25°, 24.593°, 26.892°, 29.809°, and 30.327° were corresponding and were confirmed by the Joint Committee on Powder Diffraction Standards, file no. 06–0362, which clearly represent the crystalline nature of SeNPs. The present study agreed with Ghaderi et al. [59] and Hussain et al. [60], who reported crystalline SeNPs prepared from plant extracts. Fardsadegh and Jafarizadeh [61] prepared SeNPs from aloe vera leaf extract. FTIR spectroscopy showed that the phytosynthesized NPs possess peaks at 1635.52 cm^−1^ and 3454.3 cm^−1^. The peak in the region between 1600 and 1700 cm^−1^ confirmed the presence of amide group, while the peak between 2900 and 3200 cm^−1^ represented O–H vibrations. Alvi et al. [62] stated that the different functional groups obtained through FTIR analysis of SeNPs synthesized by *M. azedarach* help in the reduction of biosynthesized SeNPs.

### 4.2. Effect of SeNPs on Fungal Growth Inhibition

The application of nanometals in plant disease management is promising as an alternative to chemical pesticides. The present study reported the strong antifungal activity of phytosynthesized SeNPs. Gunti et al. [56] used the SeNPs produced by *Emblica officinals* as an anti-dandruff shampoo to treat fungal infections; also, Shahverdi et al. [63] found that SeNPs fabricated by *K. pneumonia* reduced the fungal growth against *Malassezia sympodialis*, *Aspergillus terreus*, and *Malassezia furfur*. Furthermore, Kazempour et al. [64] found that SeNPs with MIC levels of 200 and 250 µg/mL inhibited *Aspergillus brasiliensis* and *Candida albicans* growth, respectively. The antimicrobial mechanism of NPs was briefed by DNA damage and cell wall disruption. NPs electrostatically interact with the cell wall or cell membrane, causing cell wall disruption. Therefore, large molecules pass through the cell membrane and destroy DNA, which causes cell death [65,66,67,68]. In addition, Shenashen et al. [69] reported that nanoparticles disturbed the fungal cell membrane and leakage in fungal cells, which caused hypha malformation and cell death. Our findings are similar to the results of Yilmaz et al. [70], which used SeNPs synthesized using tarragon extract and reported a similar inhibition zone diameter within the range of 17–36 mm against *Aspergillus niger*. The zone of inhibition of *M. azedarach-* synthesized SeNPs was in the range of 7.467 mm–18.60 mm and compared with the positive control exhibiting a ZOI of 11.233 mm.

### 4.3. Effect of SeNPs on Plant Morphological Parameters

The findings of the present study reveal that SeNPs increase growth parameters i.e., plant height, fresh and dry weight, leaf area, and root length. Our investigation found that low concentrations of SeNPs significantly boost both the control and fungal inoculation treatments’ growth parameters. The findings of the present study were in accordance with previous studies. According to Germ et al. [71], applying SeNPs in low doses has positive benefits and improves plants’ resistance to stress. Similarly, Bao-shan et al. [72] reported that exogenously administered SiO2 NPs to *Larix olgensis* seedlings under abiotic stress boost the plant height, root development, root length, and leaf fresh and dry weights. Boldrin et al. [73] also reported that selenium at trace levels encourages plant development. The current results are similar to those of Siddiqui et al. [74], who applied SeNPs to the germination of barley and concluded that SeNPs increased the growth attributes. Similarly, the findings of Chernikova et al. [75] revealed that SeNPs promoted plant height. The low concentration of SeNPs increases plant growth.

### 4.4. Effect of SeNPs on Plant Physiological Parameters

The present study reported the physiological parameters in terms of chlorophyll content and the membrane stability index. Our results demonstrated that SeNPs enhanced the biosynthesis of chlorophyll content and the membrane stability index. Selenium protects antenna complexes, which in return increases the amount of photosynthetic content [76]. The findings of this study are similar to those of Quiterio-Gutiérrez et al. [77]. Zahedi et al. [78] reported that SeNPs improved the chlorophyll content of tomato plants under *Alternaria solani* stress. Dong et al. [79] found that SeNPs increased the chlorophyll content in *Lycium chinense* leaves by 200–400%. The improvement in physiochemical activities of Se-treated Chinese cabbage subsequently increased the growth and development of plants [80], with more tubers of larger size in the case of potato crops [81,82]. The present results agreed with those of Rady et al. [83], who reported that SeNPs promote physiological attributes against *Phaseolus vulgaris*. Nasibi et al. [84] reported that pre-inoculation of fox tail seeds with SeNPs under stress significantly increased physiological parameters as compared to plants grown under salinity stress without seed priming. The high uptake of water by plants causes an increase in chlorophyll content [85,86]. Similarly, with a decline in oxidative stress, NPs boost the plant photosynthetic activity [87,88]. Our findings are also similar to the reports of Nasirzadeh et al. [89], who described that SeNPs increased the physiological parameters under cadmium stress in wheat.

### 4.5. Effect of SeNPs on Plant Biochemical Parameters

In the present study, the different concentrations (10, 20, 30, and 40 mg/L) of phytosynthesized SeNPs enhanced the biochemical parameters (i.e., proline, soluble sugar, phenolics, and flavonoids) of wheat under control conditions and under biotic stress of spot blotch disease. Proline helps in the protection of plants from oxidative damage and in the maintenance of an osmotic environment. [90]. Proline also protects proteins against denaturation when they are exposed to harsh circumstances [91]. Similarly, soluble sugar is considered an essential organic solute and is used for cell homeostasis. Sugar content also affects the plant’s immune system by acting as a signal molecule interacting with hormone signaling [92]. Phytosynthesized SeNPs improved wheat plants’ proline and sugar content under the stress of spot blotch disease, according to the current data from the field experiment. The findings are consistent with those of Tripathi et al. [93], who found that 20 mgL^−1^ of SeNPs increased osmolyte synthesis in strawberry plants under stress circumstances. The findings of Nasirzadeh et al. [89] reported SeNPs to increase the physiological and biochemical parameters under cadmium stress in wheat. Likewise, Sardar et al. [94] reported that plants raised from seed primed with SeNPs enhanced the proline and soluble sugar contents. The findings of current research work were nearly equal to Rady et al. [83] by applying SeNPs exogenously to improve the antioxidant defense system of *Phaseolus vulgaris*. The present study supports the findings of prior research [60,83,95]. It appears that SeNPs increased proline synthesis by increasing the activity of nitrate reductase, which is necessary for proline synthesis [96]. According to Shahid et al. [97], selenium increases the production of soluble sugars by improving the cytoplasmic membrane’s integrity and decreasing malondialdehyde, which promotes overall growth. In addition, our findings are consistent with El-Hoseiny et al. [95], who used SeNPs against mango malformation and reported that soluble sugar contents increased due to foliar application of SeNPs.

Plants are subjected to various biotic stressors, which alter their electron transport system, which participates in the production of reactive oxygen species [98]. Plants’ natural response to stress is the synthesis of non-enzymatic antioxidants such as phenolic and flavonoid compounds [99]. Phenols serve as substrates for numerous antioxidant enzymes and free radical scavengers [100]. Weintraub et al. [101] reported that reactive oxygen species (ROS), particularly phenolic chemicals, inhibit penetration, limit fungal growth, and cause tissue necrosis and cell death, all of which would stop future fungal progress toward plant tissue. Due to their significant ability to control the generation of free radicals, the application of NPs tends to enhance ROS, which activates flavonoids as an antioxidant defense mechanism [102]. Many studies have found that using various NPs can create antioxidant chemicals, which can help plants resist pathogens. Spot blotch disease reduced the levels of non-enzymatic chemicals in wheat plants, according to our findings. This is because spot blotch diseases have the most lethal impact on plants’ antioxidant defense mechanisms. However, our findings demonstrated that applying SeNPs to spot blotch-affected plants increased the synthesis of flavonoids and phenolic compounds compared with untreated wheat plants. The results of the present study agreed with the previous studies [83,95,103]. The current findings are consistent with those of Quiterio-Gutiérrez et al. [77]. Similarly, the current findings support the findings of Lopez-Vargas et al. [104], who found that CuNPs raised 36.14% flavonoids in tomatoes. Shahraki et al. [105] reported that the application of nano-Se significantly increased the antioxidant activity of leaves and flowers under non-saline (30 and 4%) and saline (12 and 22%) conditions compared to the control, respectively. Guleria et al. [106] reported the strong antioxidant activity of SeNPs. Previous studies by Kondaparrthi et al. [107], Mellinas et al. [108], Boroumand et al. [109], and Dumore et al. [110] reported the strong antioxidant activity of green-synthesized SeNPs. The results of current research work regarding flavonoids and phenolic content were in line with those reported by Hussein et al. [103].

### 4.6. Advantages of Using Green Nanotechnology over PGPR

In this period of climate change and resource limitation, challenges to crop production in terms of biotic and abiotic stress are considerable. Managing these challenges with conventional agrochemicals is no longer practical, as they will significantly and negatively impact the environment and human health. Hence, sustainable and innovative approaches are essential to successfully counteract the adverse impacts of biotic and abiotic stress. Plant growth-promoting rhizobacteria (PGPR) are heterogeneous root-associated beneficial bacteria which are known for their ability to enhance plant growth through either direct or indirect phyto-stimulatory mechanisms. PGPR reduces the deleterious effects of phytopathogens and protects the plant against biotic and abiotic stress conditions [111]. However, the variability in the performance of PGPR under varied climates, weather parameters, and soil characteristics is a significant difficulty in exploring its field efficacy. PGPR formulations are applied as suspensions to seeds, root surfaces, or soil [112]. It is difficult for a single microbial inoculant to perform consistently under varying agro-climatic conditions and stresses; therefore, recent trends in PGPR application adopt multiple inocula. Microbial consortia have proven to have higher efficiency than the application of a single species [113]. Maintenance of adequate growth conditions over time in terms of nutrition and climate are major hurdles in transferring the developed consortia from the lab to the field. Failure to maintain the desired environs can considerably affect microbial counts, which can adversely affect field results. Hence, introducing innovative and effective methods for the field delivery of PGPR is important [114,115]. To overcome this problem of PGPR, nanotechnology can be used. Nanotechnology is an emerging field that offers tremendous applications in all aspects of science. The application of nanotechnology in the agricultural sector has gained immense attention due to its ability to enhance biotic and abiotic stress tolerance, disease detection and prevention, and refined nutrient absorption. Nanomaterials can improve the nutrient utilization efficiency of plants when compared to conventional approaches. Nanoparticles (NPs) can boost plant metabolism through their defined physicochemical properties [116].

## 5. Conclusions

The current study described that phytosynthesized SeNPs could induce resistance against *B. sarokiniana* disease-susceptible wheat varieties. Under in vitro study, SeNPs also inhibit the growth of *B. sarokiniana*, showing the great antifungal potential over commonly used and harmful fungicides. Wheat plants under the stress of spot blotch disease responded positively, both physiologically and biochemically, to 30 µg/mL foliar application of SeNPs. As a result, plant morphological, physiological, and biochemical attributes were improved. SeNPs-induced resistance was due to the activation of plant defense-related enzymatic and non-enzymatic content. This resistance was due to the production of proline, phenolic, and flavonoid content. It is believed that biogenic SeNPs have ecofriendly and biocompatible relations over fungicides that have a negative impact on the environment. The cost of these NPs is suitable for farmers. Therefore, SeNPs with a concentration of 30 mg/L may be a potent antifungal agent to control spot blotch disease in wheat and other fungal diseases in plants.

## Figures and Tables

**Table 1:** Overall experimental layout.

Treatments	Concentrations (mg/L)
To	Control
T1	Control+ 10 mg/L SeNPs
T2	Control + 20 mg/L SeNPs
T3	Control + 30 mg/L SeNPs
T4	Control + 40 mg/L SeNPs
T5	Fungus Inoculated Wheat
T6	Pathogen + 10 mg/L SeNPs
T7	Pathogen + 20 mg/L SeNPs
T8	Pathogen + 30 mg/L SeNPs
T9	Pathogen + 40 mg/L SeNPs

**Table 2:** A rating scale for the leaf spot blotch disease.

Number	Symptoms Level	Resistant Level
0	No symptoms	Resistant
1	1–5% of spot on the leaves	Moderately Resistant
2	6–20% of spot on the leaves	Moderately Resistant
3	21–40% of spot on the leaves	Moderately susceptible
4	41–60% of spot on the leaves	Moderately susceptible
5	Above 61% spot on leaves	Susceptible

## Data Availability

All data is included in the manuscript.

## Supplementary Materials

The following are available online at https://www.mdpi.com/article/10.3390/plants12040761/s1, Figure S1: Green Synthesis of Bio fabricated SeNPs (a) Stock solution, (b) Plant Extract, (c) Mixed stock solution + Plant Extract and (d) brick red color of solution confirmed the formation of SeNPs; Figure S2: isolation and microscopic identification of *Bipolaris sarokiniana* (a) infected leaves were collected, (b) Emergence of fungus on culture plate, (c) Purification of fungus, and (d) Showing conidia under microscope.; Figure S3: Antifungal Activity.
