# Supplementary material for: Evaluation of Selenium Nanoparticles in Inducing Disease Resistance against Spot Blotch Disease and Promoting Growth in Wheat under Biotic Stress
Source: Plants (Basel). 2023 Feb 8;12(4):761. doi: 10.3390/plants12040761 (PMC9958785; doi:10.3390/plants12040761)
Supplement: Supplementary file 1 [file plants-12-00761-s001.zip › plants-2025630-supplementary.pdf]

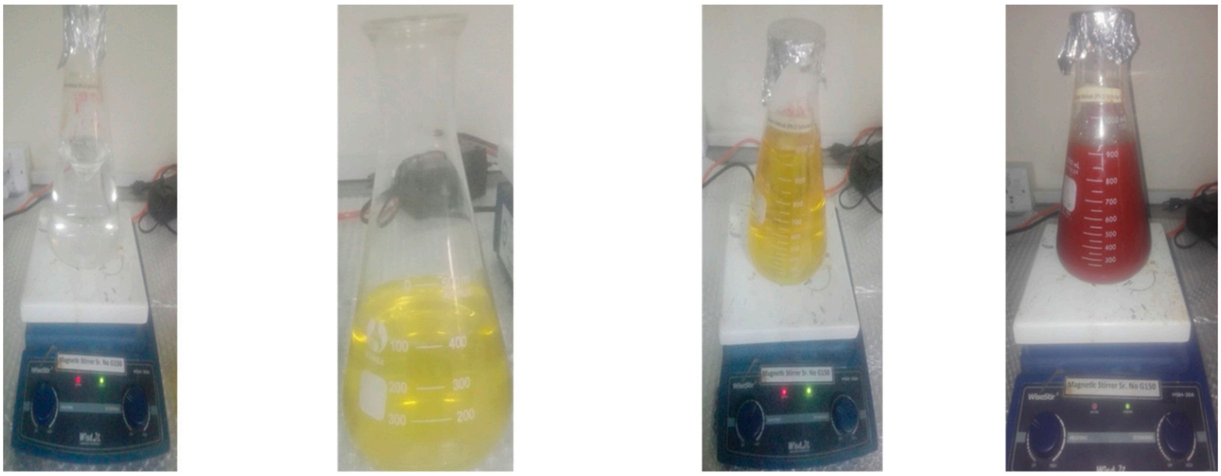

(a) (b) (c) (d)

Supplementary Figure S1: Green Synthesis of Bio fabricated SeNPs (a) Stock solution, (b) Plant Extract, (c) Mixed stock solution + Plant Extract and (d) brick red color of solution confirmed the formation of SeNPs

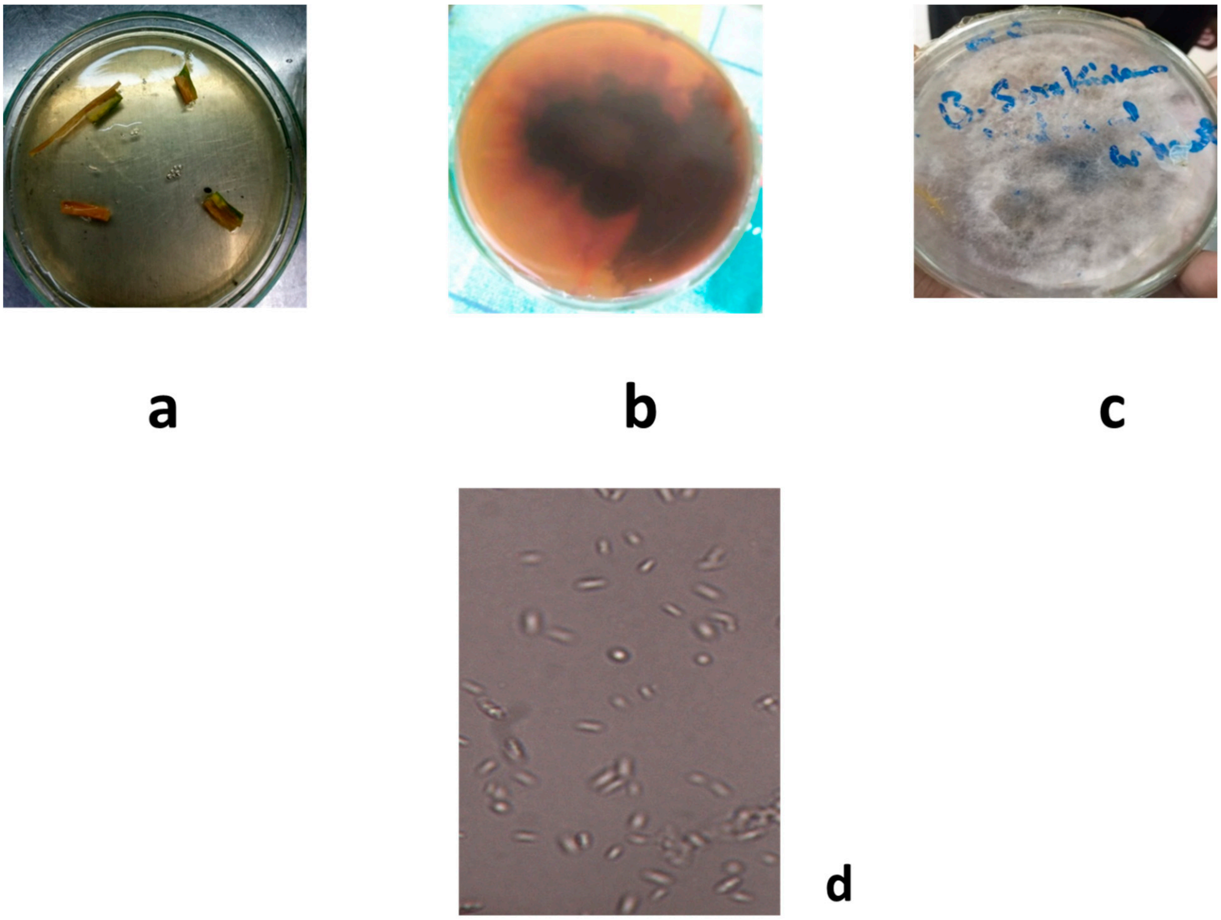

Supplementary Figure S2 :isolation and microscopic identification of *Bipolarissarokiniana* (a) infected leaves were collected, (b) Emergence of fungus on culture plate, (c) Purification of fungus, and (d) Showing conidia under microscope.

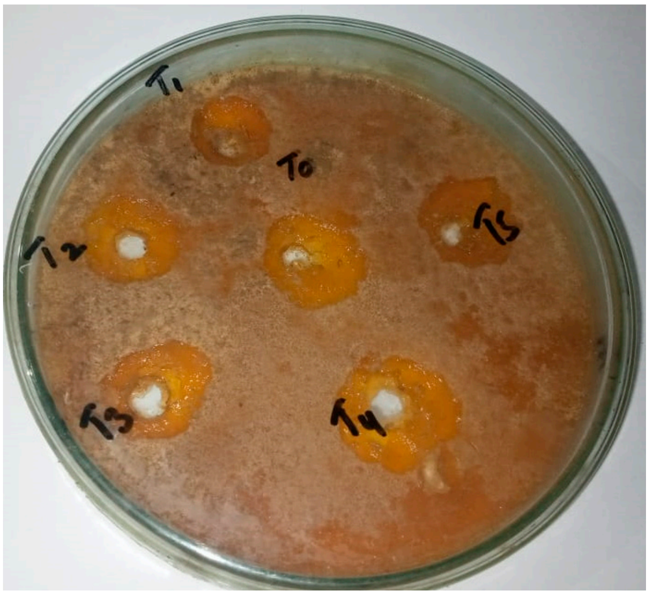

Supplementary Figure S3 : Antifungal Activity
